# Ultraconfined terahertz phonon polaritons in hafnium dichalcogenides

**DOI:** 10.1038/s41563-025-02345-0

**Published:** 2025-09-15

**Authors:** Ryan A. Kowalski, Niclas S. Mueller, Gonzalo Álvarez-Pérez, Maximilian Obst, Katja Diaz-Granados, Giulia Carini, Aditha Senarath, Saurabh Dixit, Richarda Niemann, Raghunandan B. Iyer, Felix G. Kaps, Jakob Wetzel, J. Michael Klopf, Ivan I. Kravchenko, Martin Wolf, Thomas G. Folland, Lukas M. Eng, Susanne C. Kehr, Pablo Alonso-Gonzalez, Alexander Paarmann, Joshua D. Caldwell

**Affiliations:** 1 Interdisciplinary Materials Science Graduate Program, Vanderbilt University, Nashville, TN USA; 2 Department of Physical Chemistry, Fritz Haber Institute of the Max Planck Society, Berlin, Germany; 3 Department of Physics, Freie Universität Berlin, Berlin, Germany; 4 Department of Physics, University of Oviedo, Oviedo, Spain; 5 Istituto Italiano di Tecnologia, Arnesano, Italy; 6 Institute of Applied Physics, Technische Universität Dresden, Dresden, Germany; 7 ct.qmat - Excellence Cluster TU Dresden-Würzburg, Dresden, Germany; 8 Vanderbilt University, Department of Mechanical Engineering, Nashville, TN USA; 9 Department of Physics and Astronomy, University of Iowa, Iowa City, IA USA; 10 Institute of Radiation Physics, Helmholtz-Zentrum Dresden Rossendorf, Dresden, Germany; 11 Oak Ridge National Laboratory, Oak Ridge, TN USA

**Keywords:** Polaritons, Terahertz optics, Nanophotonics and plasmonics, Two-dimensional materials

## Abstract

The confinement of electromagnetic radiation to subwavelength scales relies on strong light–matter interactions. In the infrared and terahertz spectral ranges, phonon polaritons are commonly employed to achieve deeply subdiffractional light confinement, with such optical modes offering much lower losses in comparison to plasmon polaritons. Among these, hyperbolic phonon polaritons in anisotropic materials offer a promising platform for light confinement. Here we report on ultraconfined phonon polaritons in hafnium-based dichalcogenides with confinement factors exceeding *λ*_0_/250 in the terahertz spectral range. This extreme light compression within deeply subwavelength thin films is enabled by the large magnitude of the light–matter coupling strength in these compounds and the natural hyperbolicity of HfSe_2_. Our findings emphasize the role of light–matter coupling for polariton confinement, which for phonon polaritons in polar dielectrics is dictated by the transverse–longitudinal optical phonon energy splitting. Our results demonstrate transition-metal dichalcogenides as an enabling platform for terahertz nanophotonic applications.

## Abstract (web summary)

The authors demonstrate deeply subwavelength light confinement in the terahertz spectral range by exploiting the strong light–matter coupling and hyperbolicity of phonon polaritons in hafnium-based dichalcogenides.

## Main

A prominent focus of the field of nanophotonics is the ability to confine electromagnetic energy to highly subdiffractional length scales. This enables increased electric field intensities that can enhance light–matter interactions. Remarkable light compression has been achieved by reducing the light–matter interaction volume using resonant cavities and atomic-layer thicknesses of materials^1,2^. Nonetheless, this increased optical confinement is severely hampered in many cases owing to high losses. Polaritons, hybrid light–matter quasiparticles, have facilitated extreme light confinement^3–6^, thus enabling subdiffractional imaging^7,8^, nanoscale spectroscopy^9^ and nanophotonic circuits^10^. Strong optical responses from exciton polaritons occur at visible and near-infrared wavelengths, whereas extremely confined plasmon polaritons in atomically thin graphene form at longer wavelengths^6^. At mid-infrared (mid-IR) wavelengths, optically active polar lattice vibrations couple with light to create phonon polaritons (PhPs) that can propagate with high momenta **k** (refs. ^11,12^), spectral tunability^13^ and propagation directionality^14^ with the advantage of substantially reduced losses with respect to plasmon polaritons in conductors^11,12^.

Surface PhPs can occur within the *Reststrahlen* band (RB) of polar dielectric materials, which is defined as the frequency range between the transverse optical (TO) and longitudinal optical (LO) phonons, within which the real part of the dielectric permittivity tensor is negative (*ε* < 0)^11,12^. The modes comprise evanescent waves bound to the surface of the polaritonic medium with subdiffractional polariton wavelengths *λ*_p_. Moving from bulk to thin films, the surface-bound waves hybridize and confine further (~*λ*_0_/30, where *λ*_0_ is the free-space wavelength)^15,16^ as the thickness decreases (proportional to 1/*d*, where *d* is the thickness)^17,18^. In the same vein, efforts to achieve maximum confinement (*λ*_0_/*λ*_*p*_) have been focused on using atomically thin films^1,18^ or identifying anisotropic materials that exhibit an extreme form of birefringence called hyperbolicity^18–20^, which occurs in materials with dielectric permittivities of opposite sign along different crystallographic directions. In hexagonal crystals, such as hexagonal boron nitride (hBN), hyperbolicity is realized along in- and out-of-plane crystallographic directions resulting in *ε*_∥_*ε*_⊥_ < 0. Hyperbolicity is commonly found in van der Waals (vdW) crystals, which comprise covalently bonded two-dimensional (2D) sheets along the tangential plane (*x–y*, labelled as t) that are stacked axially (*z*) and held together by weaker vdW bonds^3–5,8^. Phonons in uniaxial vdW crystals with polarizations oriented in the tangential and axial directions are, therefore, not degenerate and induce a natural anisotropy resulting in non-identical permittivities (*ε*_t_ ≠ *ε*_z_). The large confinement occurs owing to the extraordinary modes, namely hyperbolic PhPs that have nominally unrestricted magnitudes of **k** that traverse through the volume of the material^18^. Furthermore, in ultrathin hyperbolic films, where the thickness is far below the free-space wavelength (*d* « *λ*_0_), the PhP confinement scales as 1/*d* down to the monolayer limit^18^. Despite reports of exceptionally high confinement (*λ*_0_/500)^1^, the losses encountered at these atomic scales far outweigh the intensity required for a PhP to propagate sufficiently for many applications. At more modest film thicknesses (*d* = 10–100 nm), PhP propagation is achievable but not without sacrificing confinement to values of approximately *λ*_0_/100 (refs. ^18,20^). Similar confinement levels have also been achieved with an alternative approach of placing a thin high-index superstrate on an isotropic medium that supports PhPs^16,21^.

PhP materials with high confinement, such as alpha-phase molybdenum trioxide (α-MoO_3_)^14,20,22^ and hBN^5,18^, have large LO–TO phonon splitting (>100 cm^−1^)^18,23^. Broad RBs play an important role in confinement, as the large bandwidth allows the PhP to extend to high **k** before dissipating while maintaining a non-zero group velocity (*v*_g_ = d*ω*/d**k**). Furthermore, the magnitude of the light–matter coupling strength is directly related to the LO–TO phonon energy splitting, which is a function of the Born effective charge^11^. However, this is also an absolute quantity that tends to be larger for mid-IR phonons compared with those resonant within the terahertz range. A polar dielectric material with a similar RB width as hBN at terahertz frequencies would, therefore, exhibit drastically increased light–matter coupling strengths and stronger optical confinement than other terahertz resonant materials^19,24–26^. Exceptionally large Born effective charges of hafnium-based dichalcogenides (HfDCs) give rise to their large LO–TO energy splittings^27,28^, whereas the heavy Hf-ion mass places their natural optical phonon frequencies in the terahertz range. The natural anisotropy of these 2D vdW crystals, accompanied with their strong light–matter coupling strength, highlights them as excellent candidates for exploring ultraconfined PhPs in the terahertz spectral regime^27^.

In this work, we observe ultrahigh confinement of terahertz light within two HfDCs, hafnium disulfide (HfS_2_) and hafnium diselenide (HfSe_2_), with PhP wavelengths *λ*_p_ that are strongly compressed with respect to the free-space values (*λ*_0_/*λ*_p_ > 250). We examine the terahertz near-field response of this unique set of materials using a scattering-type scanning near-field optical microscope (s-SNOM), with the light provided by a free-electron laser (FEL). Although they have the same hexagonal crystal structure, HfS_2_ and HfSe_2_ have different infrared dielectric permittivities^27^ that separate the predominant types of PhPs that can be supported into elliptic and hyperbolic, respectively. In this regard, elliptic refers to an anisotropic response whereby the in- and out-of-plane dielectric permittivities are different in magnitude but are both negative in sign, whereas for hyperbolicity, one of these directions is negative while the other is positive^5^. Interestingly, we observe very strong confinement of PhPs in ultrathin films of both materials for extremely small film thicknesses *d* compared with the long terahertz wavelengths (*λ*_0_*/d* ≈ 10^3^). This is possible because the LO–TO splittings of HfS_2_ and HfSe_2_ are on par with high-confinement mid-IR PhP materials. However, when normalized by the resonant frequency *ω*_TO_, the HfDCs display much larger light–matter coupling strengths, which are responsible for the observed ultrahigh-**k** PhPs. Furthermore, we demonstrate terahertz hyperlensing by placing a metal nano-antenna below a hyperbolic HfSe_2_ flake, which enables the observation of hyperbolic rays that can be used for deeply subdiffractional terahertz imaging. The observation of ultraconfined terahertz polaritons in Hf-based vdW crystals therefore demonstrates high promise for deeply subdiffractional terahertz nanophotonic components.

## Ultraconfined thin-film polaritons

**Fig. 1:**
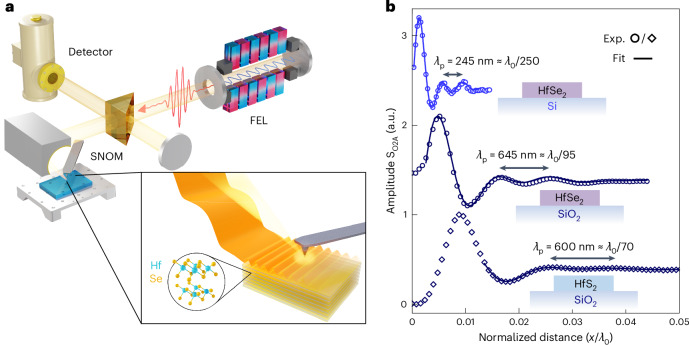
Real-space imaging of terahertz PhPs. **a**, Schematic of the experimental s-SNOM apparatus. A tunable FEL generates narrowband terahertz radiation, which is focused onto the s-SNOM tip and is backscattered into a photoconducting detector. Inset: terahertz light scatters off the edge of a HfDC flake, launching PhPs, which are then coupled out to free space by the s-SNOM tip. The layered crystal structure of vdW-bonded HfSe_2_ is also shown. **b**, Line profiles, normalized by the free-space wavelength *λ*_0_, of the raw s-SNOM amplitude (S_O2A_, offset for clarity) show the propagation of edge-launched PhPs (with the edge to the left) for three samples: HfSe_2_ on Si (*d* = 47 nm, *λ*_0_ = 61.7 µm, blue circles), HfSe_2_ on SiO_2_ (*d* = 68 nm, *λ*_0_ = 60.7 µm, dark blue circles) and HfS_2_ on SiO_2_ (*d* = 68 nm, *λ*_0_ = 41.7 µm, dark blue diamonds). Each profile was fitted (Supplementary Information, section 2) to extract the polariton wavelengths *λ*_p_, which are labelled along with the corresponding confinement factors. Exp., experimental data.

Directly observing highly confined PhPs in the far field might be challenging owing to the intrinsic momentum mismatch with free-space light, which is orders of magnitude when comparing the terahertz range with the nanoscale polariton wavelengths supported. Near-field microscopy using s-SNOM is ideally suited to access and directly image highly confined PhP modes^29–31^. Notably, the accessible momentum range in this work is experimentally limited, rather than limited by the material response, yet still the nanotip size used for s-SNOM provides access to even larger confinement factors in the terahertz than is possible in the mid-IR. We use a tunable FEL to generate narrowband terahertz radiation^32–34^, which when scattered off the s-SNOM tip or the edges of the HfDC flakes results in scattered fields with momenta sufficient to launch and detect highly confined PhP modes. An image of the PhP field propagation was obtained by rastering the tip across the surface of the flake and collecting the scattered light at each point. Schematics of the s-SNOM apparatus and of PhPs launching from the edge of a flake are provided in Fig. 1a.

One-dimensional line profiles are extracted from the s-SNOM images (Supplementary Information, section 1) perpendicular to the edge of the flakes and plotted in Fig. 1b for three sample configurations: HfS_2_ on a SiO_2_ substrate (dark blue diamonds), HfSe_2_/SiO_2_ (dark blue circles) and HfSe_2_/Si (blue circles). Each line profile is normalized on the *x* axis by *λ*_0_. Fits of the line profiles show that edge-launched polaritons dominate the response. These are then scattered to the far field by the s-SNOM tip (Supplementary Information, section 2). The peak-to-peak distance between fringes, therefore, corresponds to the polariton wavelength^23,35^. Deeply subdiffractional modes are observed in HfS_2_/SiO_2_ (*λ*_p_ = 600 nm, *λ*_0_ = 41.7 μm and *d* = 68 nm) and HfSe_2_/SiO_2_ (*λ*_p_ = 645 nm, *λ*_0_ = 60.7 μm and *d* = 68 nm). The PhP confinement factors (*λ*_0_/*λ*_p_) are 70 and 95, respectively, as shown in Fig. 1b. Notably, we observe an even larger confinement of >250 for HfSe_2_/Si with a polariton wavelength of 245 nm (*λ*_0_ = 61.7 μm and *d* = 47 nm, blue curve in Fig. 1b). Such extreme PhP confinement, without enhancement from nano-resonators, has previously been reported only for mono- and few-layer materials with high propagation losses^1^. However, the thicknesses *d* of the HfDC flakes explored here are of the order of tens of nanometres, thus preserving long polariton propagation.

**Fig. 2:**
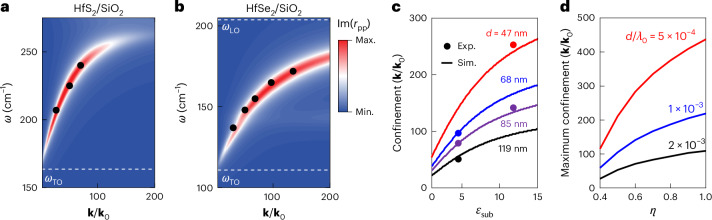
Confinement and dispersion of thin-film polaritons. **a**,**b**, PhP dispersions of HfS_2_/SiO_2_ (*d* = 68 nm) (**a**) and HfSe_2_/SiO_2_ (*d* = 68 nm) (**b**) thin films. Experimental data (black circles) are plotted over the simulated Im(*r*_pp_) illustrating the extremely large **k** of the propagating PhPs. Dashed lines show the in-plane TO and LO phonon frequencies, *ω*_TO_ and *ω*_LO_, respectively, as reported previously^27^. **c**, Light confinement of the PhPs in HfSe_2_ as a function of substrate permittivity *ε*_sub_. Confinement factors are calculated using the transfer matrix method with the flake thicknesses (*d* = 47, 68, 85 and 119 nm) used in the experimental s-SNOM imaging. Experimental values (circles) for HfSe_2_/SiO_2_ (*ε*_sub_ = 4 and *ω*_0_ = 165 cm^−1^) and HfSe_2_/Si (*ε*_sub_ = 12 and *ω*_0_ = 162 cm^−1^) are plotted along with the simulated values (solid lines, *ω*_0_ = 163.5 cm^−1^) for different HfSe_2_ thicknesses (for the colours, see the labels). **d**, Maximum confinement of thin-film polaritons of a type-II hyperbolic model material as a function of normalized light–matter coupling strength *η* at three fixed ratios of thickness to the free-space wavelength (*d/λ*_0_ = 2 × 10^−3^, black; 1 × 10^−3^, blue; and 5 × 10^−4^, red). See Supplementary Information, section 5 for details. Max., maximum; Min., minimum; Sim., simulated data.

Ultrahigh-**k** PhPs are typically observed only in hyperbolic materials, and the ultraconfined modes observed in HfSe_2_ are, indeed, hyperbolic. However, we also observe high-**k** propagating PhPs in HfS_2_ (*λ*_0_/70), which is elliptic in this spectral range^27^. To further understand such large confinement of both elliptic and hyperbolic PhPs, we examine the differences between polariton dispersions of HfS_2_ and HfSe_2_ based on experiments and simulations. For this purpose, we collected s-SNOM images at several excitation frequencies (*ω*_0_ = *c***k**_0_), where *ω*_0_ and **k**_0_ are the free-space frequency and momentum, respectively, and plot the energy versus momentum dispersions, where **k**/**k**_0_ = *λ*_0_/*λ*_p_ (Fig. 2a,b). The experimental dispersions (black circles) are in good agreement with the simulated imaginary component of the *p* polarized reflectivity Im(*r*_pp_), which was calculated using the transfer matrix method^36^ based on the dielectric permittivities extracted from far-field experiments^27^. A dispersive mode is visible for both HfS_2_/SiO_2_ (Fig. 2a) and HfSe_2_/SiO_2_ (Fig. 2b), with the dispersions extending between 160–275 cm^−1^ and 120–190 cm^−1^, respectively. The two PhPs similarly start dispersing at lower frequencies before curving towards larger values of **k**. There, however, the hyperbolic mode maintains a finite slope as well as its strong Im(*r*_pp_) intensity, in contrast to its elliptic counterpart, which decays in intensity owing to higher losses and shifts towards a near-flat dispersion. Considering the long terahertz wavelengths and the nanoscale sample thicknesses, in both cases we are firmly in the ultrathin-film regime (*d* « *λ*_0_), where the elliptic surface PhPs hybridize to create a symmetric mode and an antisymmetric mode^15,37^. The symmetric mode, commonly referred to as epsilon-near-zero, generally exhibits a flat dispersion and exists at higher frequencies, whereas the antisymmetric mode (Fig. 2a), like plasmonic thin-film modes^38^, propagates with high **k** in thin films^15^. In the following, we focus on the antisymmetric mode of HfS_2_ because it has a dispersion with a finite slope that is very much like that of hyperbolic HfSe_2_ (compare Fig. 2a,b). The dispersion of the symmetric mode, instead, could be engineered with a superstrate that leads to strongly confined polaritons with a negative group-velocity dispersion^16^^,21^ (Supplementary Information, section 7).

The momenta of thin-film PhPs depend on the thickness, the polaritonic medium permittivity, and the substrate *ε*_sub_ and superstrate *ε*_sup_ permittivities^15^^,16,18,21,39,40^ (Supplementary Information, section 5). Our experimental data (Fig. 2c, circles) show an inverse relation between thickness and confinement for several flakes of HfSe_2_ (*d* = 47 nm, 68 nm, 85 nm or 119 nm), in agreement with the simulations (Fig. 2c, solid lines). The most highly confined PhPs (*λ*_0_/*λ*_p_ > 250) that we could resolve in our experiments were observed for the thinnest flake of HfSe_2_ on a Si substrate (*ε*_sub_ ≈ 12) at a frequency *ω*_0_ = 162 cm^−1^, because the polariton confinement increases with substrate permittivity *ε*_sub_, in addition to the inverse scaling with film thickness (Fig. 2c). This also opens a route towards active tunability with phase change materials^21,41^. There is a limit, however, to how thin a film can be while still supporting the propagation of PhPs, which is defined as a PhP travelling at least one full wavelength before its amplitude decays by a factor of 1/e. At comparable thicknesses to our experiments, the maximum confinement of PhPs in mid-IR materials (for example, hBN and α-MoO_3_) plateaus around *λ*_0_/100 (refs. ^18,20^). Pushing to even thinner films does produce larger confinement^1^, but for mid-IR wavelengths, this approaches the atomic limit where propagation losses are too large for us to observe PhP modes. It is, therefore, interesting, instead, to use larger free-space wavelengths, rather than decreasing the film thickness, while still maintaining a small ratio between the two (*d*/*λ*_0_ « 1), making the terahertz range an ideal spectral range for testing the limits of ultraconfined PhPs. The key parameter for observing such ultraconfined thin-film polaritons at finite film thickness is the exceptionally large light–matter coupling of HfDCs in the terahertz spectral range. The light–matter coupling is quantified by the Rabi splitting (2*g*) between the upper and lower bulk polariton branches^42^ (Supplementary Information, section 5). These bulk PhPs exist at frequencies outside the RB, in contrast to the thin-film polaritons studied in this work, and are the result of ultrastrong coupling between infrared light and the infrared-active phonons of polar dielectrics^43^. On increasing the coupling strength *g*, the RB broadens, and the dispersion of the thin-film polaritons can extend out to larger momenta leading to larger confinement. To further generalize this phenomenon across different spectral ranges, we here employ the normalized light–matter coupling strength *η* that normalizes the Rabi splitting by the TO phonon frequency (*ω*_TO_):^43^

1 $$\eta =\frac{g}{{\omega }_{{\rm{TO}}}}=\frac{\sqrt{{\omega }_{{\rm{LO}}}^{2}-{\omega }_{{\rm{TO}}}^{2}}}{2{\omega }_{{\rm{TO}}}}.$$

To analyse how *η* affects the polariton confinement, we simulate the PhP dispersion including propagation losses of a theoretical, type-II hyperbolic material. We calculate the maximum confinement (**k**/**k**_0_ at Re(**k**) = Im(**k**)), which is where propagation losses start to dominate, thus preventing propagation. We vary *η* by changing the LO–TO splitting while keeping the frequency of maximum confinement approximately constant (Supplementary Information, section 5). The resulting achievable confinement increases with *η*, as shown for three different thickness-to-wavelength ratios in Fig. 2d, and reaches the largest values for the thinnest films. Notably, both HfS_2_ and HfSe_2_ exhibit exceptionally large values of *η* ≈ 0.8, which explains the extreme confinement observed in our experiments. More generally, it is the combination of light–matter coupling strength, hyperbolicity and thickness-to-wavelength disparity that strongly enhances the confinement of polaritons in thin films.

## Terahertz hyperlensing

**Fig. 3:**
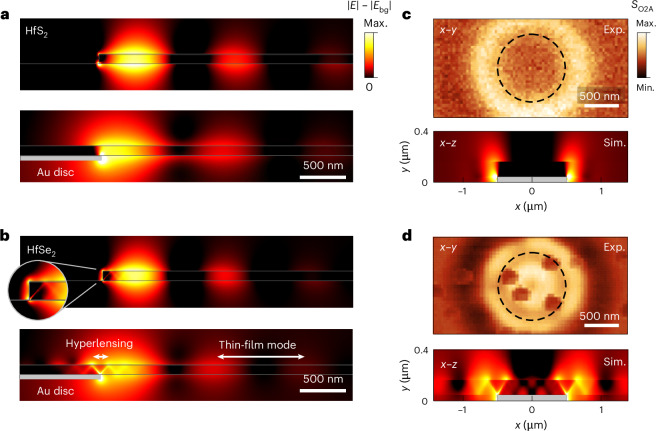
Highly subdiffractional imaging using hyperlensing. **a**,**b**, Simulated, background-subtracted electric field magnitude of PhPs in thin films (*d* = 100 nm) of HfS_2_ (**a**) and HfSe_2_ (**b**) on SiO_2_ substrates. *E* is the electric field and *E*_bg_ the background field without Au discs and HfDCs. The PhP waves are excited by a parallel-polarized plane wave at 45° incidence angle with frequencies *ω*_0_ = 240 cm^−1^ (**a**) and *ω*_0_ = 165 cm^−1^ (**b**). The waves are scattered at the sharp edge of the thin film (top panels) or by a Au disc underneath the film (bottom panels). White double-headed arrows highlight different types of propagation giving rise to hyperlensing and thin-film modes. The propagation pattern of the thin-film mode arises from the constructive and destructive interference with the plane wave. Inset: zoom-in of a hyperbolic ray launched from the flake edge. **c**, Experimental top-view (*x–y*) s-SNOM images (top, *ω*_0_ = 240 cm^−1^) and simulated side view (*x–z*) of the electric field magnitude (bottom, *x–z*) for HfS_2_ in a hyperlensing configuration. A thin film of thickness 115 nm is placed on top of a Au disc of radius *r* = 500 nm. **d**, Similarly, s-SNOM images and simulated electric fields for HfSe_2_ in a hyperlensing geometry (*ω*_0_ = 168 cm^−1^).

Beyond the extremely compressed wavelength of polaritons observed here, hyperbolic materials can additionally support higher-order modes whose superposition leads to diffractionless, ray-like polaritons travelling at distinct frequency-dependent angles within the volume of the crystal^8,44^ (Supplementary Information, sections 4 and 6). These effects, however, depend on the specific experimental geometry as to whether these polariton rays can contribute impactfully to the near-field images, as illustrated in Fig. 3. We illustrate the contrast in mode profiles by simulating the electric fields for elliptic and hyperbolic PhPs in thin films of HfS_2_ (Fig. 3a) and HfSe_2_ (Fig. 3b), respectively. We compare edge-launching for HfDC thin films placed on a SiO_2_ substrate (Fig. 3a,b, top) as well as launching by a Au disc (Fig. 3a,b, bottom), which acts like a nano-antenna that scatters incident light much more efficiently than the thin-film edge. The elliptic thin-film mode in HfS_2_ is launched by the edge of the thin film and the Au disc with identical wavelengths and similar profiles. The hyperbolic PhPs in HfSe_2_, launched by the thin-film edge (Fig. 3b, top), are dominated by the fundamental (M0) mode and very closely resemble their elliptic counterparts in this ultrathin-film regime. However, unlike in HfS_2_, we clearly resolve hyperbolic rays in HfSe_2_ when placed on the Au disc, which constitute the superposition of all higher-order modes^45^. We note that a weak ray can be also seen without the Au disc substrate, which is due to direct but less efficient excitation of these modes from the thin-film edge (Fig. 3b, top, inset). The thin-film edge, thus, mainly launches the fundamental M0 mode, which is visible in Fig. 1b. We note that there is also a narrow elliptic band in HfSe_2_ (~120–140 cm^−1^). However, this range is dominated by losses, and we were unable to observe PhPs (Supplementary Information, section 6).

The ray-like propagation of hyperbolic polaritons enables nanoscale hyperlensing and image magnification, owing to the rigid angle of polariton ray propagation. By employing HfDCs in a hyperlensing geometry, we experimentally demonstrate terahertz hyperlensing in Fig. 3c,d refs. ^7,8^. Thin flakes of HfS_2_ (Fig. 3c, *d* = 115 nm) and HfSe_2_ (Fig. 3d, *d* = 115 nm) were transferred onto a Au disc (*r* = 500 nm and *h* = 40 nm) and imaged with s-SNOM. The near-field patterns measured on the surfaces of the HfS_2_ (Fig. 3c, top, *ω*_0_ = 240 cm^−1^) and HfSe_2_ (Fig. 3d, top, *ω*_0_ = 168 cm^−1^) consist of bright ‘hot rings’ encircling the circumference of the Au disc (Fig. 3c,d, top, dashed circles), consistent with similar measurements reported for hBN^7,8^. However, another ring appears on the HfSe_2_ sample.This ring is within the circumference of the Au disc, indicating that, indeed, we observe terahertz hyperlensing for hyperbolic HfSe_2_ (see Supplementary Information, section 6 for frequency-dependent data and hyperlensing angles). We note that the dark spots in the s-SNOM intensity of HfSe_2_ (Fig. 3d, top) emerge from oxidation defects that are common for transition-metal selenides^46^, but interestingly, these do not seem to influence the polariton propagation by scattering or other deleterious interactions.

To corroborate these experimental observations, we show the simulated electric fields from a 2D side-view profile (*x–z*, Fig. 3c,d, bottom) for both cases, elliptic HfS_2_ and hyperbolic HfSe_2_. The Au disc scatters the incident light and strongly focuses the electric fields around the sharp edges, subsequently launching PhPs into the HfDC thin film above. For elliptic HfS_2_ at that frequency, only the antisymmetric thin-film mode is supported, and thus, the blurry ring corresponds to the first lobe of that wave and is observable only owing to the thin-film nature of the layer. By contrast, for hyperbolic HfSe_2_, the gold edge couples not only to the fundamental M0 but also to higher-order modes to form hyperbolic rays^8,44^ that propagate radially in both directions from the edge of the Au disc. The simulations confirm that, indeed, our results show hyperlensing in the terahertz range in a natural crystal, here indicating the imaging of a 1 μm-diameter particle, which is ×60 smaller than the free-space wavelength (59.5 μm).

## Outlook and conclusion

**Fig. 4:**
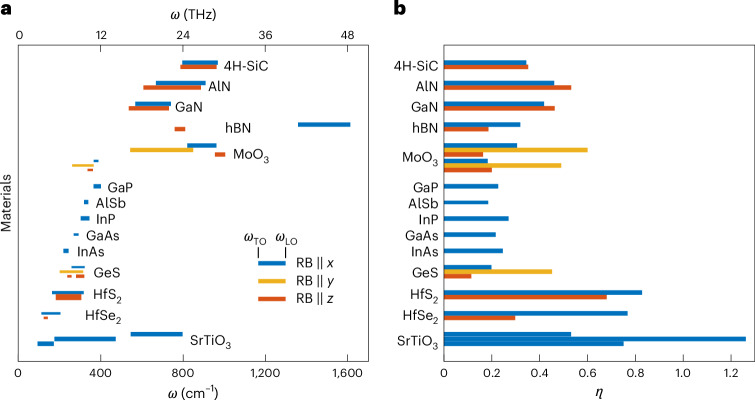
Light–matter coupling strengths of mid-IR and terahertz polar dielectrics. **a**, RBs (*ω*_TO_ to *ω*_LO_) of several polar dielectric materials in the terahertz to mid-IR range. Anisotropic materials are represented by different colour bands, with isotropic materials by one band (blue), uniaxial materials by two bands (blue and red) and biaxial materials by three bands (blue, red and yellow). **b**, Normalized coupling strength *η* calculated with equation (1) for each material in **a**.

Shrinking electromagnetic waves to subdiffractional mode volumes is essential for the development of nanophotonic devices, enhancing imaging capabilities and exploring nanoscale phenomena. In the infrared, the mechanisms to achieve high-**k** PhPs have primarily been reserved to identifying hyperbolic media or pushing light confinement to the absolute limit^18,35^. The observation of ultrahigh-**k**, broadband, elliptic and hyperbolic PhPs in HfS_2_ and HfSe_2_ in the terahertz range represents a considerable addition to the library of polaritonic materials. Moreover, it reveals the importance of the light–matter coupling strength, a material property that strongly influences the propagation of polaritons. The search for PhP materials with ultrastrong light–matter coupling strengths has largely been focused on the mid-IR, where polar dielectrics such as silicon carbide (SiC)^47^, α-MoO_3_ (ref. ^23^) and III nitrides^18,48,49^ display large LO–TO phonon splittings (>100 cm^−1^). A notable exception is SrTiO_3_, which has broad RBs from the terahertz to the mid-IR and, thus, provides an opportunity to explore ultraconfined PhPs in oxide membranes^26^. The RBs of several PhP materials are shown in Fig. 4a, which illustrates the difference in size between those in the mid-IR and terahertz. However, to fully understand the coupling strength of a polaritonic material, it is necessary to consider the magnitude of its phonon splitting relative to the TO phonon frequency. After calculating *η* for the same set of dielectrics (Fig. 4b), we find those values for HfS_2_ and HfSe_2_ to be nearly double those of most previously reported mid-IR materials, despite their smaller absolute RB widths. These large coupling strengths are the result of the exceptionally large Born effective charge magnitudes and the low TO frequencies^27,28^.

Despite the interest in the terahertz community regarding nanophotonic applications such as biomolecule fingerprinting^50^, medical diagnostics^51^ and thermal management^52,53^, traditional optics have restricted the spatial resolution of imaging and spectroscopy owing to the long free-space wavelengths. Furthermore, compressing electromagnetic fields to subdiffractional length scales results in high intensities, which introduces nonlinearities that may facilitate terahertz strong-field physics^54^. In this work, we imaged in real space ultrahigh-**k** terahertz PhPs (*λ*_0_/*λ*_p_ > 250) in 2D vdW crystals where the thickness does not approach the atomic limit (≥50 nm). The high normalized light–matter coupling strengths of HfDCs, which facilitate large confinement while avoiding deleterious losses, are highlighted as a crucial parameter that aids the identification of novel polaritonic materials. We have found that it is feasible to propagate terahertz light at the length scale of visible wavelengths, thus helping to push the field of nanophotonics to its limits.

## Methods

### Sample fabrication

Thin flakes were fabricated by mechanical exfoliation of HfSe_2_ and HfS_2_ bulk crystals (HQ Graphene) using the Scotch tape method^55^. Flakes were exfoliated onto either Si with an ~1-μm-thick oxide layer, which served as the SiO_2_ substrate, or high-resistivity float zone Si.

### Hyperlens fabrication

Hyperlensing samples were fabricated by deterministically transferring 2D flakes of HfS_2_ and HfSe_2_ onto Au discs using a polymer-assisted transfer method. The discs were fabricated on a Si substrate using electron-beam lithography and resistive Au deposition. The discs had a thickness of 40 nm. HfDC flakes were mechanically exfoliated onto a Si substrate, removed using a polydimethylsiloxane stamp and transferred onto the Au discs.

### FEL-SNOM

Polariton imaging was performed using an s-SNOM from Attocube Systems. The microscope is attached to the free-electron laser facility FELBE at the Helmholtz-Zentrum Dresden-Rossendorf, Germany^33,34^. The s-SNOM tip, with tapping frequency *Ω* ≈ 160 kHz, focuses the FEL radiation at its apex and scatters the signal into the far field. The scattered signal is composed of several harmonics (*nΩ*, *n* = 1, 2, 3, …), which are demodulated from the linear far-field background. A liquid helium-cooled gallium-doped germanium photoconductive detector (QMC Instruments Ltd) was used to detect the scattered signal. For the edge-launched polaritons, the second harmonic near-field signal (S_O2A_, *n* = 2) was recorded using either homodyne or self-homodyne interferometric detection, and the hyperlensing measurements were made using self-homodyne detection^56^. The experiments were conducted with a FEL because of its fully tunable and narrowband radiation. Future measurements of these materials could be conducted with narrowband table-top terahertz lasers at selected frequencies or with broadband terahertz light sources.

### Transfer matrix simulations

The dispersion maps shown in Fig. 2 were calculated using a transfer matrix algorithm^36^. We evaluated the imaginary component of the parallel-polarized reflection coefficient for evanescent wave excitation at a given in-plane momentum **k**. Peaks in these maps correspond to absorptive polariton resonances^36^, whereas the linewidth of these peaks in horizontal cuts (at constant frequency) relate to the propagation losses and the linewidth in vertical cuts (at constant momentum) relate to the polariton lifetime. These quantities were used to estimate the maximum achievable confinement, as shown in Fig. 2d (see Supplementary Information, section 5 for details).

### Full-wave finite-element simulations

Full-wave finite-element simulations were conducted with COMSOL Multiphysics using the electromagnetic waves, frequency-domain solver of the RF Module. A 2D simulation cell in the *x–z* plane (vertical cross section through the HfDC film) was constructed to simulate the hyperlensing or edge-launching experiments. Plane-wave illumination (normal incidence for hyperlensing) was used to excite PhPs. The simulation cell was surrounded by perfectly matched layers. We used the dielectric function of HfDCs from ref. ^27^ and set *ε*_∞,z_ = 7 for HfSe_2_ for the hyperlensing simulation in Fig. 3d. The background electric field without the Au disc launcher or the HfDC was subtracted.

## Online content

Any methods, additional references, Nature Portfolio reporting summaries, source data, extended data, supplementary information, acknowledgements, peer review information; details of author contributions and competing interests; and statements of data and code availability are available at 10.1038/s41563-025-02345-0.

## Supplementary information

Supplementary Information Supplementary Sections 1–7.

## Data Availability

All data that support the findings of this study are present in the main text and the Supplementary Information. All raw data generated during the current study are available from the corresponding authors upon request.

## Code availability

The code used for the transfer matrix method in this study to calculate the polariton dispersions is available via Zenodo at 10.5281/zenodo.601496 (ref. ^57^).
