# Supplementary material for: Ultraconfined terahertz phonon polaritons in hafnium dichalcogenides
Source: Nat Mater. 2025 Sep 15;24(11):1735–41. doi: 10.1038/s41563-025-02345-0 (PMC12575359; doi:10.1038/s41563-025-02345-0)
Supplement: Supplementary file 1 — Supplementary Sections 1–7. [file 41563_2025_2345_MOESM1_ESM.pdf]

nature materials

Supplementary information https://doi.org/10.1038/s41563-025-02345-0

Ultraconfined terahertz phonon polaritons in hafnium dichalcogenides

In the format provided by the authors and unedited

Contents

Section S1. s-SNOM Polariton Imaging 2
Section S2. Polariton Wavelength Fitting and Extraction 4
Section S3. Polariton Wavelength versus Flake Thickness 4
Section S4. Thin-Film Polariton Propagation in Hyperbolic vs Elliptic Material 5
Section S5. Maximum Confinement of Thin Film Phonon Polaritons 6
Section S6. Hyperlens Images 11
Section S7. Engineering the Dispersion of Thin-Film Polaritons with a Superstrate 13
References 15

Section S1. s-SNOM Polariton Imaging

We perform s-SNOM polariton imaging on three sets of flakes: HfS_2 on a SiO_2 substrate, $HfSe_2$ on a SiO_2 substrate, and $HfSe_2$ on a Si substrate. For each set, we measured three or four different flakes with thicknesses: $HfSe_2/SiO_2$ (d = 68, 85, and 119 nm), $HfSe_2$/Si (d = 47, 75, and 85 nm), and HfS_2/SiO_2 (d = 68, 112, 200, and 400 nm). Using a tunable free-electron (FEL) laser, we measure at several frequencies (ω) within their respective Reststrahlen bands. Near-field images and line profiles of HfS_2/SiO_2 (Figure S1a-c, d = 68 nm), $HfSe_2/SiO_2$ (Figure S1d-h, d = 85 nm), and $HfSe_2$/Si (Figure S1i-n, d = 47 nm) flakes are measured at the edge of the flakes where phonon polaritons are launched due to scattering of the incident FEL radiation. Polaritons propagate into the flake perpendicular to the edge, as seen by the oscillating s-SNOM amplitude (S_{O2A}). Due to the long THz wavelengths and extremely large polariton confinement, interference of the free-space wavelength with the polaritons is not expected.

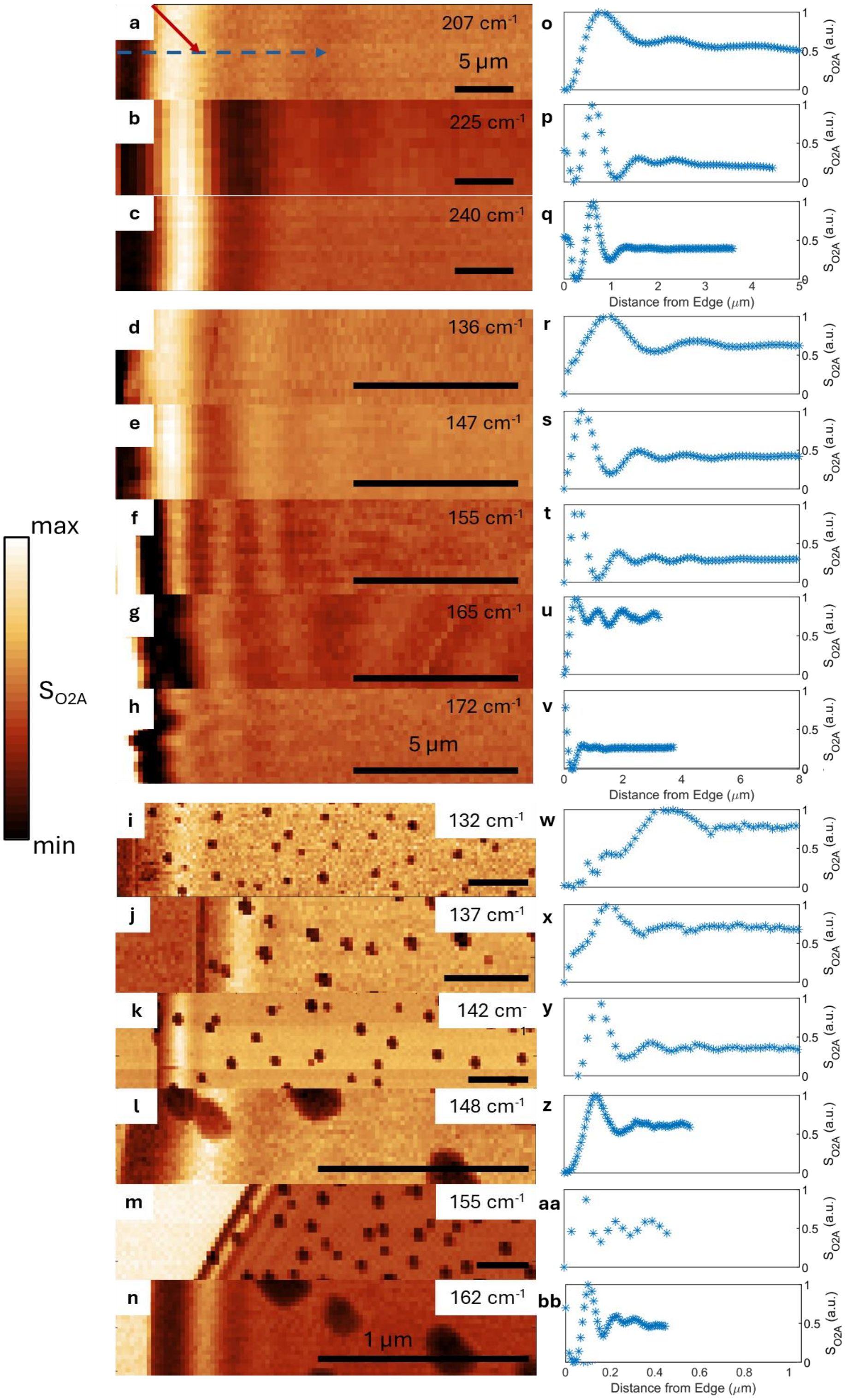

Figure S1. *Representative near-field optical amplitude images of the same flake at several frequencies of (a-c) HfS_2/SiO_2 (d = 68 nm), (d-h) $HfSe_2/SiO_2$ (d = 85 nm), and (i-n) $HfSe_2/Si$ (d = 47 nm), respectively. Scale bars are 5 µm, 5 µm, and 1 µm for each set of materials, respectively. Line profiles extracted from the images for (o-q) HfS_2/SiO_2, (r-v) $HfSe_2/SiO_2$, and (w-bb) $HfSe_2/Si$. A blue dashed arrow indicates the path of*

the line scans extracted in (o-bb), perpendicular to the edge of the flake and red arrow represents the direction of the incident laser.

Section S2. Polariton Wavelength Fitting and Extraction

The phonon polariton (PhP) wavelengths were extracted by fitting line profiles of the oscillatory near-field amplitude perpendicular to the flake edge. The line profiles were averaged along the length of the edge for improved signal to noise. In the case of $HfSe_2$, where inherent oxidation spots on the surface produced artifacts, a threshold filter was implemented to exclude them from the analysis. An exponentially-decaying sinusoidal function of distance from the flake edge *x*, was used to fit the near-field signal S_{opt} and extract both the PhP wavelength λ_p and the propagation distance L_p.

$$S_{opt}(x) = S_0 + A_1 \frac{e^{-\frac{2x}{L_p}} \sin\left(2\pi \frac{x - x_1}{\lambda_p}\right)}{\sqrt{x}} + A_2 \frac{e^{-\frac{x}{L_p}} \sin\left(\pi \frac{x - x_2}{\lambda_p}\right)}{x}, \quad A_1, A_2, L_p, \lambda_p > 0 \qquad \text{(S1)}$$

An offset term (S_0) was included to account for remaining background scattering signal from the far-field. The two sinusoidal terms describe the damped propagation of a circular wavefront. The first term, with amplitude A_1, phase offset x_1, and a $1/\sqrt{x}$ dependence, accounts for polariton waves that are launched from the s-SNOM tip, back-reflected by the flake edge, and scattered out by the tip, resulting in an interference between the forward- and backward propagating polaritons (tip-edge-tip). The second term, with $1/x$ dependence and phase offset (x_2), represents edge-launched polaritons that do not experience wave interference, but are directly scattered from the tip into the far-field (edge-tip). From the fits of the s-SNOM images in Figure S1 we obtained A2 >> A1, which shows that the polaritons have predominantly edge-launched character.

Section S3. Polariton Wavelength versus Flake Thickness

We plot the polariton wavelengths of all flakes as a function of flake thickness *d* in Figure S2. Each flake was scanned using at least two ω. Linear fits are applied to each act as a guide-to-the-eye.

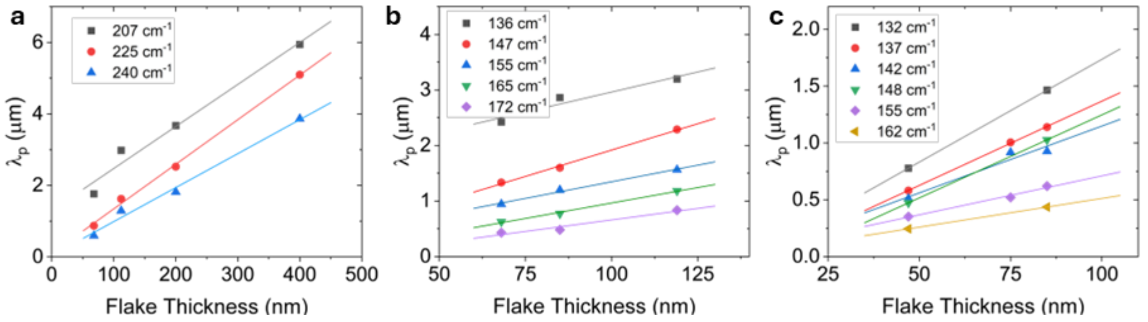

Figure S2. *Experimental polariton wavelengths (data points), extracted from line profiles of s-SNOM images (Figure S1), as a function of flake thickness for (a) HfS_2/SiO_2, (b) $HfSe_2/SiO_2$, and (a) $HfSe_2/Si$. The thickness dependence was fit for each excitation frequency (see legend). Linear fits of the data are plotted as guides to the eye (solid lines).*

Section S4. Thin-Film Polariton Propagation in Hyperbolic vs Elliptic Material

To compare the propagation of thin-film polaritons in hyperbolic $HfSe_2$ with elliptic HfS_2, we simulate propagation patterns with Comsol and correlate them with the polariton dispersion from transfer-matrix simulations (Figure S3, S4, and Figure 3a,b of the main text). The dispersions are calculated for 100 nm thick films of $HfSe_2$ or HfS_2 on a SiO_2 substrate. The dispersion of the $HfSe_2$ film is characterized by several type-II hyperbolic thin-film modes, similar to the thin-film modes that occur in the upper Reststrahlen band of hBN (Figure S3a).[1,2] For the elliptic HfS_2, instead, only one mode exists at a specific frequency (Figure S3b). Only the spectral range of the anti-symmetric elliptic thin-film mode is shown, because all measured propagation patterns in this work belong to this mode.

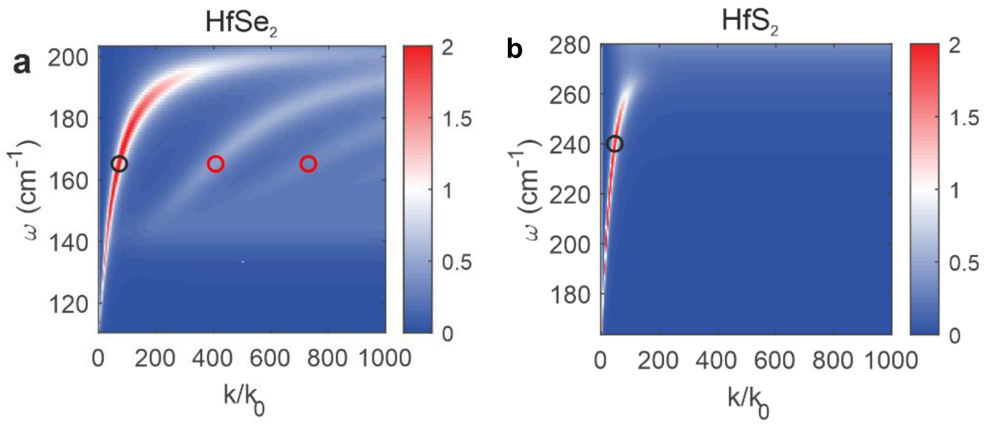

Figure S3. *Simulated dispersions of polaritons in 100 nm thick films of $HfSe_2$ (top) and HfS_2 (bottom) on a SiO_2 substrate. (a) Transfer-matrix simulation $\mathrm{Im}(r_{pp})$ of the thin-film polariton dispersion of $HfSe_2$. Fundamental and higher-order hyperbolic modes are visible and highlighted by black and red circles*

respectively at frequency of the Comsol simulations of Figure 3, main text. (b) The same as (a) but for HfS_2, which does not support hyperbolic modes and therefore only possesses a single branch.

We simulate the propagation patterns of edge-launched polaritons by illuminating an edge of the thin films with a p-polarized plane wave at 45° incidence angle (Figs. S4a-c and Figure 3a,b main text). The scales in Figs. S4a-c are normalized by the free-space wavelength, λ_0, displaying the difference in confinement between the elliptic and hyperbolic thin-film modes. Surprisingly, the propagation patterns of $HfSe_2$ and HfS_2 are very similar, despite being hyperbolic vs elliptic. This is because the edge-launching in $HfSe_2$ dominantly occurs through a coupling to the fundamental hyperbolic mode (Figure S3a, black circle), which has a similar dispersion as the elliptic thin film mode of HfS_2 (Figure S3b). The ray-like hyperbolic propagation, instead, occurs through a superposition of the fundamental hyperbolic mode with higher-order modes that have much larger momenta (Figure S3a, red circles and Figure 3 main text).[1–4] Polaritonic rays appear to be launched much more efficiently by the corner of an Au disk than the edge of a thin film, explaining the marked difference in propagation in our experiments with $HfSe_2$ thin films (Figs. 1-3, main text).

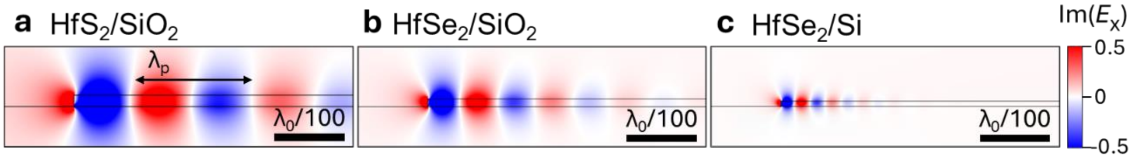

Figure S4. *Simulated electric near fields $Im(E_x)$ of edge-launched polaritons for the material-substrate combinations in Figure 1b main text, with excitation frequencies 240, 165, and 172 cm^{-1}, respectively. Scale bars are normalized to the free-space wavelength, $\lambda_0/100$.*

Section S5. Maximum Confinement of Thin Film Phonon Polaritons

We use transfer matrix calculations to analyze the role of hyperbolicity, film thickness, light-matter coupling strength, losses, dielectric screening, and substrate permittivity for the maximum achievable confinement of thin film phonon polaritons. As a general model for phonon polaritons, we use a Lorentz dielectric function

$$\epsilon_L(\omega) = \epsilon_\infty \left(1 - \frac{4\eta^2 \omega_{TO}^2}{\omega^2 - \omega_{TO}^2 + i\gamma\omega}\right), \qquad (S2)$$

with ω_{TO} the transverse optical phonon frequency, γ the materials losses, and ϵ_∞ the high-frequency dielectric constant. We use the normalized coupling strength

$$\eta = \frac{g}{\omega_{TO}} \qquad (S3)$$

to parametrize the light-matter coupling strength, independent of the frequency range of a specific phonon polariton. g is the absolute light-matter coupling strength, that corresponds to half of the Rabi splitting between the lower and upper bulk polariton branch, Figure S5. It is related to the LO-TO splitting as

$$g = \frac{\sqrt{\omega_{LO}^2 - \omega_{TO}^2}}{2}. \tag{S4}$$

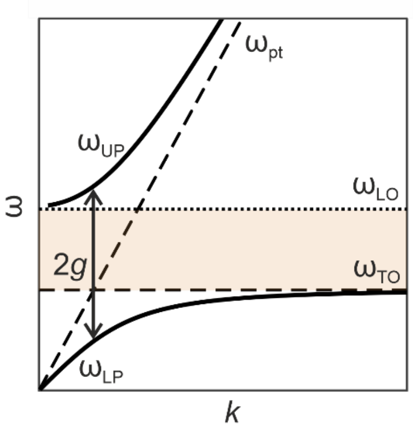

Figure S5. *Dispersion of bulk phonon polaritons with upper ω_{UP} and lower ω_{LP} polariton branch (solid lines) with minimum frequency separation $2g$ at the crossing point of transverse optical phonon ω_{TO} and photon ω_{pt} dispersions (dashed lines). The splitting of longitudinal ω_{LO} (dotted line) and transverse ω_{TO} optical phonon frequencies defines the Reststrahlenband (shaded area).*

We model a type-II hyperbolic material with a diagonal permittivity tensor, with the components $\epsilon_{xx} = \epsilon_{yy} = \epsilon_L(\omega)$, $\epsilon_{zz} = \epsilon_\infty$. An isotropic material is modeled with $\epsilon_{xx} = \epsilon_{yy} = \epsilon_{zz} = \epsilon_L(\omega)$.

Figure S6a,d show example dispersions for hyperbolic and isotropic thin-film polaritons, obtained from transfer matrix calculations of $\mathrm{Im}(r_{\mathrm{pp}})$ for different relative confinements k/k_0 and frequencies ω. As model parameters, we choose $\eta = 0.8$, $\omega_{\mathrm{LO}} = 100\ \mathrm{cm}^{-1}$, $\gamma = 5\ \mathrm{cm}^{-1}$, $\epsilon_\infty = 10$, and a film thickness of $d = 100\ \mathrm{nm}$, which are close to the experiments with $HfSe_2$ and HfS_2. For simplicity we simulate a freestanding film and discuss the role of a substrate below. For hyperbolic thin film modes, the largest confinement occurs close to ω_{LO}. For isotropic thin film modes there are two polariton branches, which correspond to a symmetric (even mode, higher frequency) and antisymmetric (odd mode, lower frequency) combination of the E_z fields of surface phonon polaritons at both interfaces.[5,6] Both modes converge to an asymptote frequency between ω_{TO} and ω_{LO}. For large ϵ_∞, the lower frequency odd mode is dominant and the asymptote is close to ω_{LO}, making the dispersion of isotropic and hyperbolic thin films very similar.

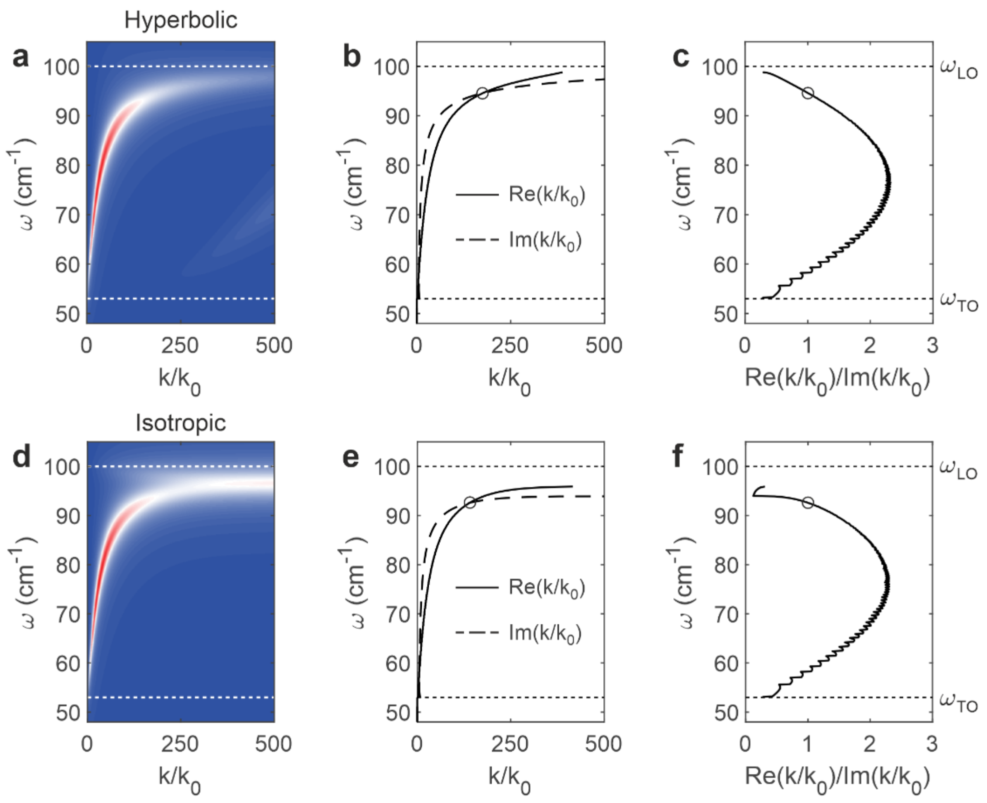

Figure S6. *Dispersion of hyperbolic (top row) and isotropic (bottom row) thin film polariton, with parameters* $\eta = 0.8$, $\omega_{LO} = 100\ cm^{-1}$, $\gamma = 5\ cm^{-1}$, $\epsilon_\infty = 10$, $t = 100\ nm$. *(a,d) Polariton dispersions from transfer matrix simulations of* $Im(r_{pp})$. *(b,e) Confinement* $Re(k/k_0)$ *and losses* $Im(k/k_0)$, *and (c,f) ratio of confinement to losses* $Re(k/k_0)/Im(k/k_0)$ *extracted from (a,d). Dotted lines show* ω_{LO} *and* ω_{TO}. *Dot shows frequency and confinement where* $Re(k/k_0) = Im(k/k_0)$.

We extract the confinement $\mathrm{Re}(k/k_0)$ and losses $\mathrm{Im}(k/k_0)$ from cuts at constant frequency $k(\omega)$, where $\mathrm{Re}(k/k_0)$ is obtained from the peak position of the fundamental thin-film mode, and $\mathrm{Im}(k/k_0)$ from its full width at half maximum (FWHM), Figs. S6b,e. The confinement and losses both increase with frequency towards ω_{LO}.

We choose $\mathrm{Re}(k/k_0) = \mathrm{Im}(k/k_0)$ as the criterion for the maximum possible confinement. In experiments this would correspond to the situation where only one fringe is visible whereas $\mathrm{Re}(k/k_0) > \mathrm{Im}(k/k_0)$ is necessary to observe polariton propagation. The ratio $\mathrm{Re}(k/k_0)/\mathrm{Im}(k/k_0)$ strongly varies with frequency and falls < 1, both, close to ω_{TO} and ω_{LO} (Figs. S6c,f). We determine the maximum confinement at $\mathrm{Re}(k/k_0)/\mathrm{Im}(k/k_0) = 1$ close to ω_{LO}, see dots in Figs. S6c,f. For the chosen parameters, the hyperbolic thin film possesses a larger maximum confinement of 175 than the isotropic thin film with 140.

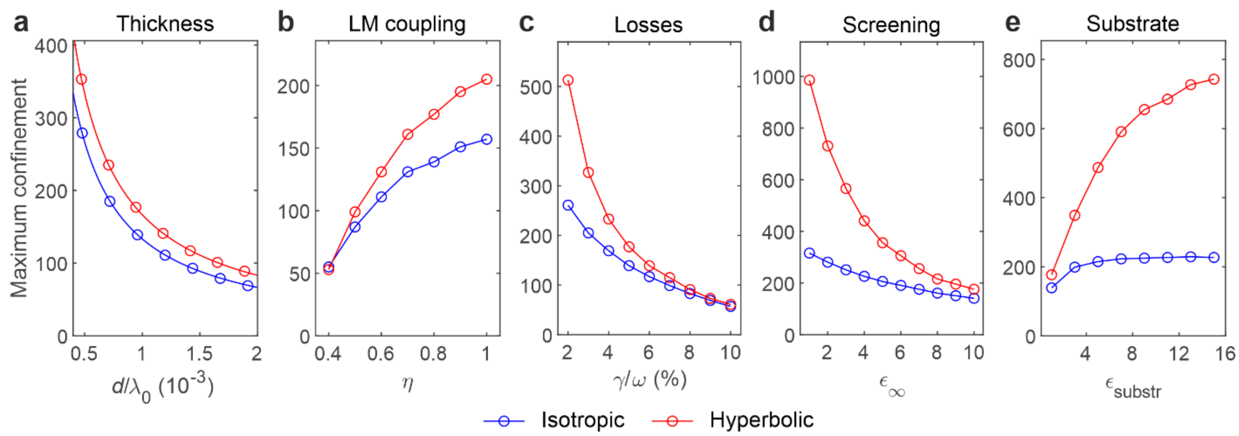

Figure S7. *Maximum confinement as function of (a) film thickness d normalized by free-space wavelength λ_0, (b) normalized light-matter coupling η, (c) losses γ normalized by frequency ω, (d) background dielectric constant ϵ_∞, and (e) substrate permittivity ϵ_{substr}, for isotropic dielectric function (blue) and hyperbolic dielectric function (red). Parameters (unless changed as shown by x axis): $\eta = 0.8$, $\omega_{LO(asymptote)} = 100\ cm^{-1}$, $\gamma = 5\ cm^{-1}$, $\epsilon_\infty = 10$, $d = 100\ nm$. Solid lines in (a) are fits with $f(d) = A/d$, with $A = const$.*

In the following we systematically study the role of hyperbolicity, film thickness d, light-matter coupling strength η, losses γ, dielectric screening ϵ_∞, and substrate permittivity $\epsilon_{\mathrm{substr}}$ for the maximum achievable confinement (Figure S7). In all calculations for type-II hyperbolic polaritons we fix $\omega_{\mathrm{LO}} = 100\ \mathrm{cm}^{-1}$, because the hyperbolic branches converge to ω_{LO} at large k. To keep ω_{LO} constant for different η, we set

$$\omega_{\mathrm{TO}} = \frac{\omega_{\mathrm{LO}}}{\sqrt{1 + 4\eta^2}} \qquad \text{(S5)}$$

in Eq. (S4). For isotropic materials we choose ω_{TO} such that the asymptote of the thin film mode occurs at $100\ \mathrm{cm}^{-1}$. The polariton frequency where the maximum confinement occurs therefore stays in the range $90 - 98\ \mathrm{cm}^{-1}$ and is approximately constant with respect to the film thickness.

We find the following trends:

- Film thickness d: The maximum confinement of, both, hyperbolic and isotropic polaritons scales as $\propto 1/d$ (solid lines in Figure S7a). [5,7]
- Light-matter coupling η increases the maximum confinement. The increase is slightly stronger for hyperbolic polaritons.
- Losses γ decrease the maximum confinement. The decrease is slightly more pronounced for hyperbolic polaritons.

- Screening ϵ_∞ decreases the maximum confinement, which is much more pronounced for hyperbolic polaritons.
- The substrate permittivity ϵ_{subtr} is a key parameter to tune the confinement of hyperbolic polaritons, while it can only slightly tune the confinement of isotropic polaritons.

Figure S8 shows transfer matrix calculations of the maximum confinement based on the dielectric functions of $HfSe_2$ and HfS_2 , instead of the model parameters in Figure S7. The material thickness is normalized to the free-space wavelength at which the maximum confinement occurs. This enables a direct comparison of both materials. We observe the same $\propto 1/d$ dependence of the maximum confinement as for the model dielectric function in Figure S7. For a freestanding film (Figure S8a) the confinement of both materials is only $\times$ 1.4 larger for $HfSe_2$. This is because both materials have similar light-matter coupling ($\eta_{\mathrm{HfSe2}} = 0.77$ vs $\eta_{\mathrm{HfS2}} = 0.83$), similar losses ($\gamma_{\mathrm{HfSe2},xy}/\omega_{\mathrm{LO}} \approx 3.9\%$ vs $\gamma_{\mathrm{HfS2},xy}/\omega_{\mathrm{LO}} \approx 3.4\%$) and similar dielectric screening ($\epsilon_{\infty,xy,\mathrm{HfSe2}} = 7.25$ vs $\epsilon_{\infty,xy,\mathrm{HfS2}} = 6.3$). The major difference is therefore the hyperbolicity of $HfSe_2$. The confinement of the hyperbolic $HfSe_2$ can be strongly increased by a substrate ($\times$ 2.4 for SiO_2 and $\times$ 3.4 for Si), while only a small increase is achieved for the elliptic HfS_2 ($\times$ 1.3 for SiO_2 and $\times$ 1.4 for Si). A similar enhancement for hyperbolic polaritons was also observed in Figure S7a. This substrate effect explains why we observe a larger confinement for the hyperbolic thin film polaritons in $HfSe_2$ in our experiments.

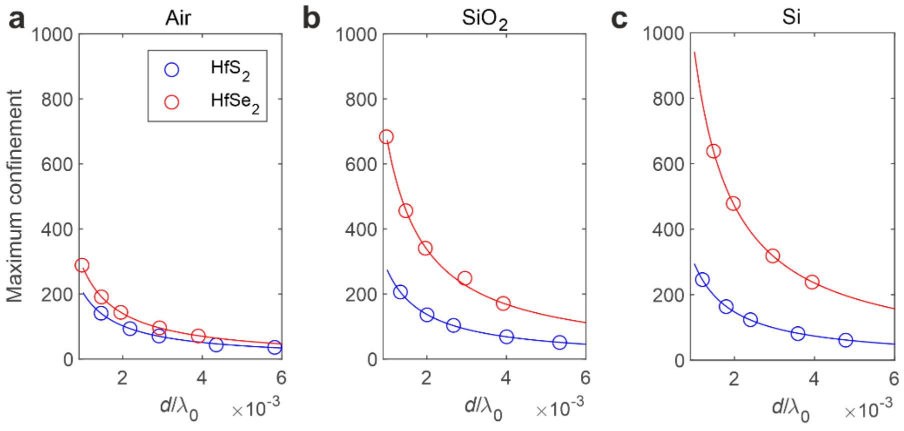

Figure S8. *Maximum confinement of thin-film phonon polaritons in $HfSe_2$ (red) and HfS_2 (blue) as function of film thickness d (a) for a freestanding film in air, (b) on a SiO_2 substrate, and (c) on a Si substrate. Solid lines are fits with $f(d) = A/d$, with $A = const$.*

Section S6. Hyperlens Images

In the hyperlensing experiment with $HfSe_2$, the gold disk placed beneath the material is expected to launch polaritonic rays that propagate with angle θ with respect to the surface normal through the material. In SNOM images, this gives rise to a double-ring feature at the surface of the material. As the angle θ varies with ω, the ring diameters are expected to change with frequency.[3,8–10] Indeed, we observe such double rings with varying separation as function of ω for $HfSe_2$, confirming its hyperbolicity (see images in Figure S9 and extracted line scans in Fig. S10a). We extract several values of $\theta(\omega)$ by imaging at multiple frequencies (Figure S9) and measuring the radius of the outer ring with respect to the edge of the disk.[3] Radial line scans of the s-SNOM intensity are first extracted by an angular averaging about the center of the disk and plotted on Figure S10a for frequencies within the hyperbolic range. Then, $\theta(\omega)$ is calculated using the following equation,

$$\theta = \arctan\left[\frac{r_{PhP} - r_{disk}}{d}\right], \qquad \text{(S6)}$$

where r_{PhP} is the radius of the outer hyperbolic PhP hot ring at the surface of the $HfSe_2$ thin film, r_{disk} (500 nm) is the radius of the Au disk launcher, and d the film thickness of 115 nm.

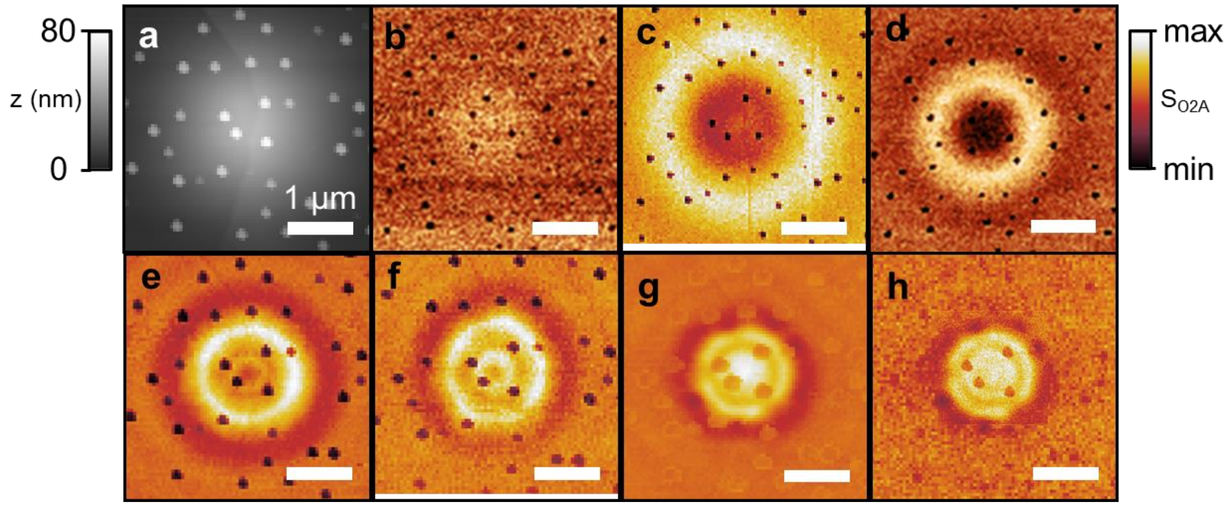

Figure S9. *Near-field s-SNOM images of $HfSe_2$ in the hyperlensing geometry shown in Figure 3d of the main text, i.e. a flake (d = 115 nm) of $HfSe_2$ on a Au disk (r = 500 nm, h = 40 nm) (a) Topographical map of $HfSe_2$ thin film on top of the Au disk. (b-h) Hyperlensing images of $HfSe_2$ at frequencies 131, 147, 159, 163, 168, 175, 182 cm^{-1}. All scale bars are 1 µm.*

The angle θ at which the hyperbolic PhP rays propagate through the volume of the material (with respect to surface normal) is defined by the arctangent of the ratio of permittivities[8]

$$\theta(\omega) = \frac{\pi}{2} - \arctan\left(\sqrt{-\frac{\mathrm{Re}(\varepsilon_z(\omega))}{\mathrm{Re}(\varepsilon_t(\omega))}}\right) \tag{S7}$$

with ε_t the in-plane and ε_z the out-of-plane permittivity. The angles extracted from experiments follow the general trend predicted by Eq. (S7), see Fig. 10b, confirming the hyperlensing by the $HfSe_2$ thin film. We find an offset between the experimental (Figure S10b, circles) and theoretical θ (dotted line), calculated using the dielectric permittivity from Ref. 11. Using the same model, we adjust ε_z by changing the out-of-plane high-frequency permittivity constant from $\varepsilon_{\infty,z} = 13.8$ as reported in Ref. 11 to $\varepsilon_{\infty,z} = 7$ (solid line) to obtain a better agreement for the frequency dispersion in θ. Indeed, such a high out-of-plane non-resonant electronic permittivity is unusual among TMDCs, where typically the in-plane exciton resonances and interband transitions are stronger than the out-of-plane, resulting in a larger non-resonant electronic in-plane permittivity in the infrared, compared to the out-of-plane value.[12] It should also be noted that FTIR micro-spectroscopy, as employed in Ref. 11 to extract the initial dielectric function, is very sensitive to optical dipoles with in-plane polarizations, but is limited in sensitivity to out-of-plane polarizations as Cassegrain objectives cannot readily surpass the Brewster angle where the out-of-plane response dominates.[13] The vertically-oriented s-SNOM tip on the other hand, introduces a corresponding polarization making it a complementary technique to far-field FTIR spectroscopy.[14,15]

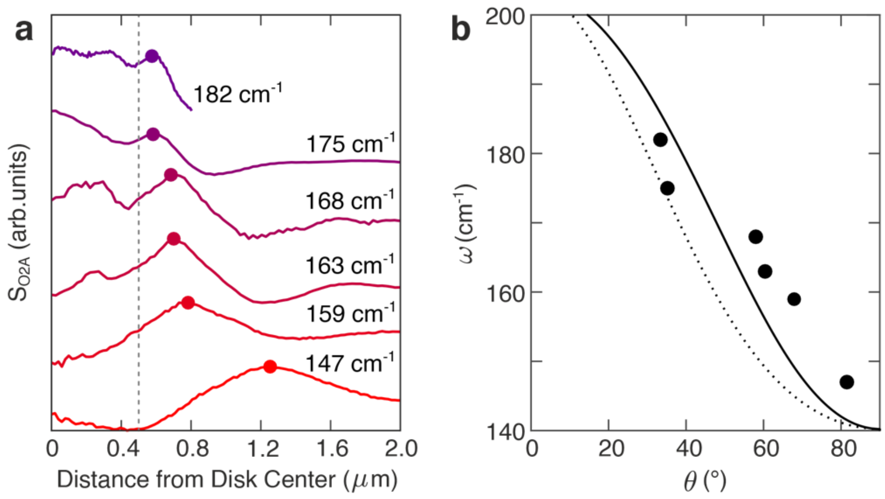

Figure S10. *(a) Radial line profiles of the hyperlensing images in Figure S9. Data points show the center of the outer ring extracted from fits. Grey dashed line shows the lateral position of the edge of the gold disk. (b) Hyperbolic propagation angle θ as a function of ω. Black circles show experimental values calculated from the data in (a). Theoretically-calculated values are plotted using the dielectric function from Ref.* 11

(dotted line) and a similar refined dielectric function with adjusted out-of-plane high-frequency permittivity $\varepsilon_{\infty,z}$ = 7 (solid line).

Section S7. Engineering the Dispersion of Thin-Film Polaritons with a Superstrate

A powerful approach to increase the confinement of surface phonon polaritons is to place a thin high-permittivity superstrate on top of an isotropic or elliptic medium.[16,17] Here, we test the applicability of this approach to thin-film phonon polaritons in elliptic HfS_2 and hyperbolic $HfSe_2$ based on transfer-matrix calculations.

In the case of elliptic HfS_2, a thin high-index superstrate changes the thin-film dispersion in an interesting way (Fig. S11). For a thin HfS_2 film (d = 100 nm) on SiO_2 there are two thin-film modes: the upper symmetric mode, often referred to as ENZ, and the lower antisymmetric mode, which is the subject of our work. Without superstrate, the symmetric mode has a very flat dispersion (Fig. S11b). The presence of a superstrate instead leads to a dispersion with negative group velocity that becomes steeper with increasing refractive index: see 10 nm of SiO_2 with $\varepsilon \approx 5$ (Fig. S11c) and 10 nm of Si with $\varepsilon \approx 12$ (Fig. S11d). In contrast, the dispersion of the lower antisymmetric mode is only weakly affected by the superstrate. A superstrate therefore offers a route to engineer the dispersion of the upper symmetric thin film mode, offering further tunability of the elliptic thin film polaritons, for example through the use of phase change materials.[16]

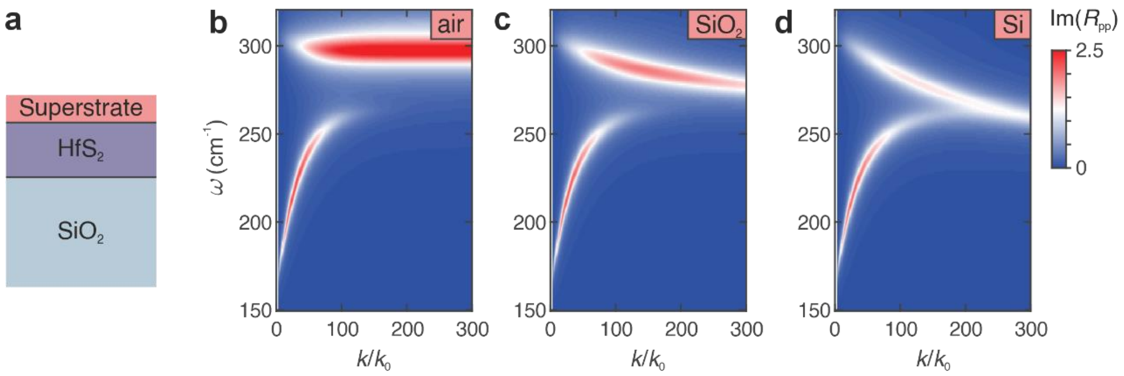

Figure S11. *Transfer matrix calculations of polariton dispersions of a 100 nm thick HfS_2 film on a SiO_2 substrate with a 10 nm thick superstrate. (a) Geometry (not to scale), (b) dispersion without superstrate, (c) dispersion with SiO_2 superstrate and (d) dispersion with Si as superstrate.*

For hyperbolic $HfSe_2$ the effect of a superstrate is very different, see Fig. S12 below. The dispersion of the M0 mode, which is investigated in our work for ultraconfined thin-film polaritons, is only merely affected by the superstrate. With increasing permittivity of the superstrate, the dispersion of the M0 mode even extends out to smaller momenta and the

maximum achievable confinement is decreased (compare Figs. S12b-d). A superstrate is therefore mostly a powerful approach to engineer the dispersion of elliptic thin-film polaritons.

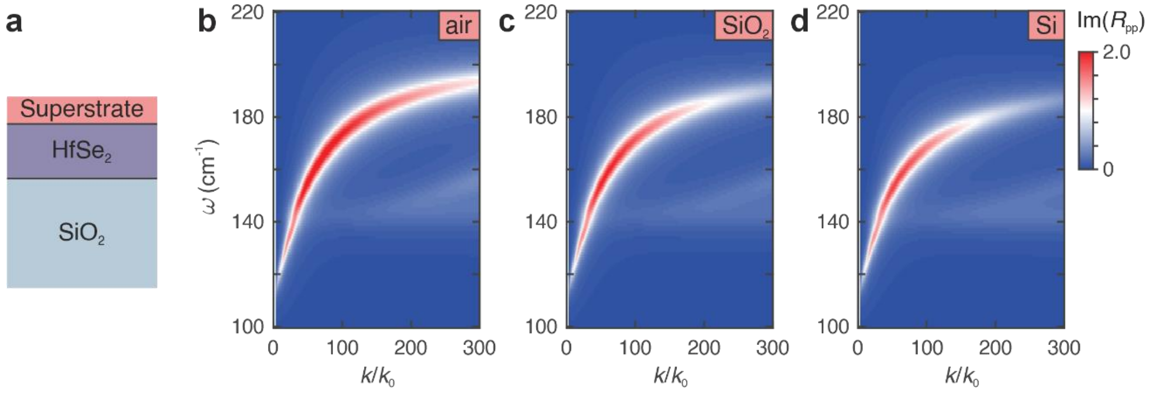

Figure S12. *Transfer matrix calculations of polariton dispersions of a 100 nm thick $HfSe_2$ film on a SiO_2 substrate with a 10 nm thick superstrate. (a) Geometry (not to scale), (b) dispersion without superstrate, (c) dispersion with SiO_2 superstrate and (d) dispersion with Si as superstrate.*

References

1. Sheinfux, H. H. *et al.* Evolution and reflection of ray-like excitations in hyperbolic dispersion media. Preprint at https://doi.org/10.48550/arXiv.2104.00091 (2022).
2. Yoxall, E. *et al.* Direct observation of ultraslow hyperbolic polariton propagation with negative phase velocity. *Nature Photon* **9**, 674–678 (2015).
3. Dai, S. *et al.* Subdiffractional focusing and guiding of polaritonic rays in a natural hyperbolic material. *Nat Commun* **6**, 6963 (2015).
4. Nikitin, A. Yu. *et al.* Nanofocusing of Hyperbolic Phonon Polaritons in a Tapered Boron Nitride Slab. *ACS Photonics* **3**, 924–929 (2016).
5. Mancini, A. *et al.* Near-Field Retrieval of the Surface Phonon Polariton Dispersion in Free-Standing Silicon Carbide Thin Films. *ACS Photonics* **9**, 3696–3704 (2022).
6. Passler, N. C. *et al.* Strong Coupling of Epsilon-Near-Zero Phonon Polaritons in Polar Dielectric Heterostructures. *Nano Lett.* **18**, 4285–4292 (2018).
7. Dai, S. *et al.* Tunable Phonon Polaritons in Atomically Thin van der Waals Crystals of Boron Nitride. *Science* **343**, 1125–1129 (2014).
8. He, M. *et al.* Ultrahigh-Resolution, Label-Free Hyperlens Imaging in the Mid-IR. *Nano Lett.* **21**, 7921–7928 (2021).
9. Li, P. *et al.* Hyperbolic phonon-polaritons in boron nitride for near-field optical imaging and focusing. *Nat Commun* **6**, 7507 (2015).
10. Jäckering, L. *et al.* Super-Resolution Imaging of Nanoscale Inhomogeneities in hBN-Covered and Encapsulated Few-Layer Graphene. *Adv. Sci.* **12**, 2409039 (2025).
11. Kowalski, R. A. *et al.* Mid- to Far-Infrared Anisotropic Dielectric Function of HfS_2 and $HfSe_2$. *Adv. Opt. Mater.* **10**, 2200933 (2022).
12. Laturia, A., Van De Put, M. L. & Vandenberghe, W. G. Dielectric properties of hexagonal boron nitride and transition metal dichalcogenides: from monolayer to bulk. *npj 2D Mater Appl* **2**, 6 (2018).
13. Folland, T. G., Nordin, L., Wasserman, D. & Caldwell, J. D. Probing polaritons in the mid- to far-infrared. *J. Appl. Phys.* **125**, 191102 (2019).
14. Álvarez-Pérez, G. *et al.* Infrared Permittivity of the Biaxial van der Waals Semiconductor α-MoO_3 from Near- and Far-Field Correlative Studies. *Adv. Mater.* **32**, 1908176 (2020).
15. F. Tresguerres-Mata, A. I. *et al.* Observation of naturally canalized phonon polaritons in LiV2O5 thin layers. *Nat Commun* **15**, 2696 (2024).
16. Li, P. *et al.* Reversible optical switching of highly confined phonon–polaritons with an ultrathin phase-change material. *Nature Mater* **15**, 870–875 (2016).
17. Dubrovkin, A. M., Qiang, B., Krishnamoorthy, H. N. S., Zheludev, N. I. & Wang, Q. J. Ultra-confined surface phonon polaritons in molecular layers of van der Waals dielectrics. *Nat Commun* **9**, 1762 (2018).
